# The Influence of Metal Stress on the Availability and Redox State of Ascorbate, and Possible Interference with Its Cellular Functions

**DOI:** 10.3390/ijms14036382

**Published:** 2013-03-20

**Authors:** An Bielen, Tony Remans, Jaco Vangronsveld, Ann Cuypers

**Affiliations:** Centre for Environmental Sciences, Hasselt University, Agoralaan Building D, Diepenbeek B-3590, Belgium; E-Mails: an.bielen@uhasselt.be (A.B.); tony.remans@uhasselt.be (T.R.); jaco.vangronsveld@uhasselt.be (J.V.)

**Keywords:** metals, oxidative stress, reactive oxygen species (ROS), ascorbate, cellular redox signal

## Abstract

Worldwide, metals have been distributed to excessive levels in the environment due to industrial and agricultural activities. Plants growing on soils contaminated with excess levels of metals experience a disturbance of the cellular redox balance, which leads to an augmentation of reactive oxygen species (ROS). Even though the increased ROS levels can cause cellular damage, controlled levels play an important role in modulating signaling networks that control physiological processes and stress responses. Plants control ROS levels using their antioxidative defense system both under non-stress conditions, as well as under stress conditions such as exposure to excess metals. Ascorbate (AsA) is a well-known and important component of the plant’s antioxidative system. As primary antioxidant, it can reduce ROS directly and indirectly via ascorbate peroxidase in the ascorbate–glutathione cycle. Furthermore, AsA fulfills an essential role in physiological processes, some of which are disturbed by excess metals. In this review, known direct effects of excess metals on AsA biosynthesis and functioning will be discussed, as well as the possible interference of metals with the role of AsA in physiological and biochemical processes.

## 1. Introduction

The metal industry and agricultural activities have led to the diffusion of metals into the environment, which has become an important process in the geochemical cycling of these elements [1]. At the end of the last century, factories in Belgium were required to switch to environmentally friendly production processes, allowing for a decrease in the amount of metals released into the environment. In many regions, former pollution still causes problems, while in developing countries, pollution still continues [2–4]. Metals can cause serious problems for all organisms when exceeding the natural emissions and through bioaccumulation from the atmosphere, soil and water. They can accumulate in crops grown on metal-contaminated soils with negative consequences for the quality and safety of feed and food crops. This in its turn is highly dangerous for food chain contamination and thus human health [1,5,6].

In all organisms, high concentrations of metals have an influence on physiological and biochemical processes, both depending on the physicochemical properties, speciation and on the dose of the metal. Essential metals such as copper (Cu), zinc (Zn), manganese (Mn), nickel (Ni), iron (Fe), *etc.* are necessary for proper plant growth, development and functioning, and these metals are toxic when present in high concentrations [7]. Non-essential metals such as cadmium (Cd), lead (Pb), aluminum (Al), metalloid arsenic (As), *etc.* are taken up by plants, despite the selectivity of transport systems, and they generate toxic responses already at low exposure concentrations [8,9].

A good knowledge of plant–metal interactions is important for reducing risks associated with the introduction of metals into the food chain [10–13]. At the morphological level, metal stress leads to visibly reduced plant growth due to the reduced cell elongation and cell wall elasticity [10,14,15]. At the molecular level, an excess of metals in plant cells can disturb the cellular redox balance and lead directly or indirectly to oxidative stress [16–18]. During this process, reactive oxygen species (ROS) are formed and thereby shift the redox balance to the pro-oxidative side. However, plant cells contain a well-equipped antioxidative defense system to attain a new balanced redox status. Because of the differences in chemical characteristics between metals, they can cause oxidative stress in a direct or indirect way. Redox-active metals such as Cu and Fe, can directly induce ROS production via Fenton and Haber–Weiss reactions [15]. In contrast, redox-inactive metals such as Cd, Ni and Zn induce ROS production only through indirect mechanisms such as inhibition of antioxidative enzymes or stimulation of ROS-producing enzymes (NADPH oxidases) [14,19]. To avoid oxidative damage, an attempt is made to maintain the increase of ROS during metal stress within physiological concentrations by the action of the antioxidative defense system [2,20,21]. This defense system consists of both enzymes such as superoxide dismutase (SOD), ascorbate peroxidase (APx) and catalase (CAT) and metabolites such as glutathione (GSH) and ascorbate (AsA) [22,23]. Although elevated levels of ROS can cause cellular damage, controlled levels of ROS play an important role in modulating signaling networks that control both physiological processes and stress responses. Hydrogen peroxide (H_2_O_2_) is a well-known example involved in the control of such processes and responses [24,25]. Ascorbate peroxidases (APx) are important scavengers of H_2_O_2_ that are responsible for protection against harmful amounts of H_2_O_2_ and for the regulation of H_2_O_2_ levels for signaling [26,27]. The enzyme APx uses AsA as a reducing agent for the conversion of H_2_O_2_ to water [28,29]. Besides the function of AsA in the regulation of defense and survival of plants, it is also involved in modulating plant growth via phytohormones. In plants, AsA is an abundant water-soluble metabolite with essential roles in multiple physiological processes [30], some of which are disturbed by excess metals. In this review, known direct effects of excess metals on AsA biosynthesis and functioning will be discussed, as well as possible interference of metals with the role of AsA in physiological and biochemical processes.

## 2. Ascorbate Biosynthesis and the Influence of Metal Exposure

### 2.1. Biosynthesis

l-ascorbic acid (ascorbate, AsA, vitamin C) is quantitatively the predominant antioxidant in plants. It is present in all subcellular compartments with an average concentration of 2 to 25 mM, or even higher in chloroplasts [31,32]. Because of this high cellular content in plants, they are the main dietary source of vitamin C for humans [32,33]. Ascorbate is also an essential compound for the plant itself, with important roles as an antioxidant and as a modulator of plant development through hormone signaling [33].

**Figure 1:**
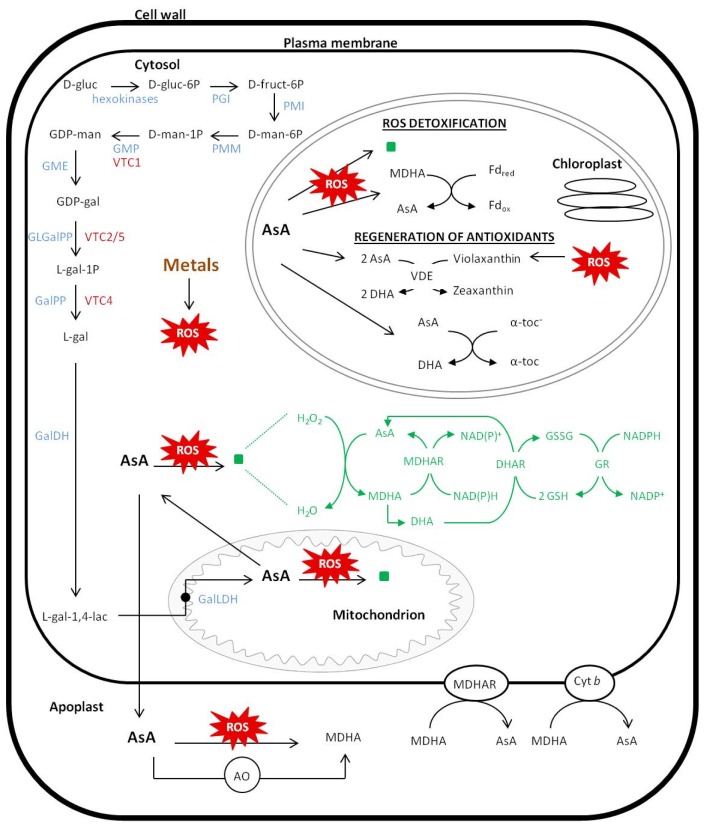
Representation of the biosynthesis, localization and antioxidant function of AsA. The biosynthesis of AsA takes place in the cytosol, except the last step occurs in the mitochondrion. Ascorbate plays a role in the antioxidant defense in two ways. One way is to scavenge ROS direct via the AsA-GSH cycle ( 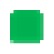) or through direct binding to ROS and produce MDHA. The other secondary manner is to regenerate antioxidants such as α-tocopherol and zeaxanthin. Abbrevations: d-gluc, d-glucose; d-gluc-6P, d-glucose-6-P; d-fruc-6P, d-fructose-6-P; d-man-6P, d-mannose-6-P; d-man-1P, d-mannose-1-P; GDP-man, GDP-d-mannose; GDP-gal, GDP-l-galactose; l-gal-1P, l-galactose-1-P; l-gal, l-galactose; l-gal-1,4-lac, l-galactono-1,4-lactone; PGI, phosphoglucose isomerase; PMI, phosphomannose isomeras; PMM, phosphomannomutase; GMP, GDP-mannose-pyrophosphorylase, GME, GDP-mannose-3′,5′-epimerase; GLGalPP, GDP-l-galactose phosphorylase; GalPP, l-galactose-1-P-phosphatase; GalDH, l-galactose dehydrogenase; GalLDH, l-galactone-1,4-lactone dehydrogenase; AsA, ascorbate; DHA, dehydroascorbate; MDHA, monodehydroascorbate; APx, ascorbate peroxidase; MDHAR, monodehydroascorbate reductase; DHAR, dehydroascorbate reductase; GSH, glutathione; GSSG, oxidized glutathione; GR, glutathione reductase; α-toc, α-tocopherol; VDE, violaxanthin de-epoxidase; Fd, ferredoxin; Cyt b, cytochrome b.

Different pathways of AsA biosynthesis have evolved in animals and plants. In animals, AsA is formed from UDP-d-glucuronate to l-gulono-1,4-lactone via d-glucuronate formation, reduction and lactonization and via oxidation of l-gulono-1,4-lactone to l-ascorbic acid [34]. In plants, the major biosynthetic pathway for AsA was discovered in 1998, and is known as the Smirnoff–Wheeler pathway (d-mannose/l-galactose pathway) (Figure 1) [35]. This pathway involves the conversion of GDP-d-mannose to GDP-l-galactose. It has been suggested that l-galactose is a widespread constituent of plant cell walls [29]. Free l-galactose has never been measured in plants, but its rapid metabolism suggests that it may be present in low quantities. l-galactose is oxidized to l-galactono-1,4-lactone, which is the immediate precursor for AsA synthesis, by a NAD-dependent l-galactose dehydrogenase (GalDH). l-galactono-1,4-lactone is oxidized to AsA via l-galactono-1,4-lactone dehydrogenase (GalLDH), which is located at the inner mitochondrial membrane and donates electrons to cytochrome C [36].

The initial three steps to d-mannose-6-P start with the conversion of d-glucose by hexokinases to d-glucose-6-P, which in turn is converted to d-fructose-6-P via phosphoglucose isomerase (PGI). The enzyme phosphomannose isomerase (PMI) that forms d-mannose-6-P has not been extensively studied in plants, although two putative genes are identified in *Arabidopsis thaliana* (*At3g02570* and *At1g67070*), based on sequence homology [37]. The activities of the next two enzymes, phosphomannomutase (PMM) and GDP-d-mannose pyrophosphorylase (GMP) in this pathway result in the formation of GDP-d-mannose [32]. As with PMI, little is known about the PMM enzyme in plants, but based on sequence homology, *At2g45790* is a candidate *Arabidopsis* PMM gene. Conklin *et al.*[38] have presented evidence that the GMP enzyme, which catalyzes the synthesis of GDP-d-mannose from d-mannose-1-P, is encoded by the *VTC1* locus (At2g39770) in *Arabidopsis thaliana*. The *vtc1-1* AsA-deficient mutant has accomplished a reduced conversion of mannose to AsA; and the activity of GMP is 30% in extracts from *vtc1-1* mutants as compared to wild-type plants [38]. These GDP-sugar intermediates are also involved in the synthesis of cell wall polysaccharides and glycoproteins [39]. The enzyme GDP-mannose 3′,5′-epimerase (GME) is known to catalyze the conversion of GDP-d-mannose to GDP-l-galactose, which is then proposed to be broken down in two steps to l-galactose. Until recently, there was a missing link between GDP-l-galactose and l-galactose. Dowdle *et al.*[40] and Laing *et al.*[41] identified the enzyme converting GDP-l-galactose to l-galactose-1-P, known as GDP-l-galactose phosphorylase (GLGalPP). They have identified the gene *VTC2* (*At4g26850*) encoding this enzyme. They tested single mutants for this gene, *vtc2-1* and *vtc2-2*, which lack an active *VTC2* gene, but still contain 10%–20% of the wildtype AsA level. Therefore, other enzymes or pathways must synthesize the remaining AsA. They identified another *Arabidopsis thaliana* gene, a homolog of *VTC2*, namely *VTC5* (At5g55120) encoding a second GLGalpp with similar properties to VTC2, and which is generally expressed at a 100–1000-fold lower than *VTC2*. The *vtc5-1* and *vtc5-2* mutants contain approximately 90% of the wild-type AsA level. They investigated their function further by constructing double mutants. Double mutants without functional *VTC2* and *VTC5* were unable to survive without AsA, showing that the GDP-mannose pathway, using GLGalPP, is the only physiologically significant source of AsA biosynthesis in *Arabidopsis thaliana* seedlings [40,41]. In addition, *VTC4* encodes a specific l-galactose-1-P phosphatase (GalPP) that contributes to the hydrolysis of l-galactose-1-P to l-galactose. The observation that *vtc4* knockout mutants are only partially deficient in AsA, as well as GalPP activity suggests that *VTC4* is not the only gene encoding an enzyme catalyzing this reaction in *Arabidopsis*[42]. The VTC2, VTC5 and VTC4 enzymes may prove to be important regulatory steps given the rapid rate of l-galactose and l-galactono-l,4-lactone conversion to AsA. The pathway prior to l-galactose is located in the cytosol, but the last step, the oxidation of l-galactono-1,4-lactone to AsA, is located in the inner mitochondrial membrane (Figure 1) [39].

The expression of some genes, determined by quantitative reverse transcription PCR, involved in AsA biosynthesis in bean (*Phaseolus vulgaris*) nodules was affected by stress conditions and particularly by Cd exposure [43]. After 26 days, the bean nodules were exposed to 100 μM CdCl_2_ for 4 days (Table 1). The mRNA levels of *GMP*, *GME*, *GalDH* and *GalLDH*, except *GalPP*, declined in Cd-exposed nodules relative to the control. Moreover, no correlation existed between *GalLDH* mRNA levels, GalLDH activity and AsA content, as the latter two remained unaffected under Cd stress. The authors suggested that the GalLDH activity in nodules is posttranscriptionally regulated in response to stress conditions [43]. This is in contrast to the study of Tamaoki *et al.*[44], where a correlation between the mRNA level of *GalLDH*, its activity and AsA content was indicated in young and mature *Arabidopsis thaliana* leaves. Such correlation was not observed in the roots, where the activity of GalLDH and AsA level were low despite a high level of *GalLDH* transcripts. It was also suggested that the expression of *GalLDH* gene may be posttranscriptionally regulated in *Arabidopsis thaliana* roots. To attain these results, total RNA was extracted from young (two-week-old) rosette leaves, mature (six-week-old) rosette leaves, inflorescence stems, flower buds, cauline leaves and roots. Furthermore, diurnal changes in AsA pool size and in the level of *GalLDH* expression were analyzed in the leaves of two-week-old seedlings, where diurnal changes in *GalLDH* transcripts, GalLDH activity and AsA content showed similar patterns [44]. Thus, the content of AsA, the activity of GalLDH and the accumulation of *GalLDH* transcripts vary with plant species or tissues.

Current evidence suggests that the Man–Gal pathway predominates in AsA biosynthesis in plants, but it is plausible that an alternative pathway also exists [31–33]. Wolucka and Van Montagu [66] have shown that biochemical conversion performed by GME not only produces the well-known GDP-l-galactose, but also GDP-l-gulose. These metabolites result from the 3′- and 5′-epimerization and the 5′-epimerization of GDP-d-mannose [31]. However, as the following steps in the branch have not yet been described in plants, it is suggested that GDP-l-gulose will be converted to l-gulose [32]. Subsequently, the conversion of l-gulose to l-gulono-1,4-lactone is catalyzed by aldonolactonase and finally, l-gulono-1,4-lactone is converted to AsA by l-gulono-1,4-lactone dehydrogenase [32,66]. Additionally, further studies are needed to clarify the role and existence of this alternative pathway in plants, as it remains to be fully proven. It will also be necessary to unravel the nature and specificity of the hydrolytic step(s) responsible for l-galactose/l-gulose release [32,66].

### 2.2. Influence of Metal Exposure on AsA Biosynthesis

The biosynthesis of AsA in plants can be influenced by metals, depending on their properties, the duration of exposure, or the specific tissue of the plant studied (Table 1). A significant increase in total AsA content was observed in different plant species under various Cd conditions, probably due to a stimulation of AsA synthesis [17,26,45,46]. In 11-day-old *Phaseolus vulgaris*, supplied with 2 μM CdSO_4_ to the roots during three days, a significant increase of the total AsA content was observed from 24 h until 72 h in the primary leaves of Cd-exposed plants [46]. In three-week-old *Arabidopsis thaliana* plants, treated with 5 or 10 μM CdSO_4_ during 24 h, increases in total AsA were observed in roots [17,26] and leaves [45]. These four studies demonstrated that, under short-term exposure of plants to the non-essential metal Cd, an increase in total AsA was observed.

Long-term exposure of Cd, next to exposure to higher concentrations, can lead to a decrease in total AsA, as shown in the studies of Schützendubel *et al.*[47] and Aravind and Prasad [48] (Table 1). In *Pinus sylvestris*[47], Cd inhibits the antioxidative systems leading to H_2_O_2_ production, followed by a transient induction of the AsA synthesis. Five-week-old *Pinus sylvestris* plants were exposed to 5 or 50 μM CdSO_4_ and root samples were collected after 6 to 96 h of exposure. In these trees, H_2_O_2_ accumulation was followed by a significant increase in total AsA after 12 h exposure of the root tips to both Cd concentrations. From this point on, a depletion of total AsA was observed in root tips exposed to 50 μM Cd for a prolonged time (>12 h) [47]. Also, Aravind and Prasad [48] reported a decrease in total AsA in *Ceratophyllum demersum* exposed to 10 μM CdCl_2_ for 1 week [48]. Thus, Cd stress leads to a decreased total AsA content due to exposure to higher concentrations or longer exposure times.

In general, exposure of plants to excess levels of essential metals such as Cu and Zn, leads to an increase in total AsA content (Table 1). In a study of Tewari *et al.*[58], mulberry (*Morus alba* L. cv. Kanva-2) plants were exposed for 25 or 50 days to 1 μM Cu used as control condition, 0 and 0.1 μM Cu (Cu-deficient conditions) and up to 100 μM Cu (excess supply). They showed that the amount of total AsA increased with increasing Cu concentrations, and Cu-deficient plants showed a decrease in the AsA content [58]. Also, other researchers observed a significantly higher total AsA content upon Cu stress. In barley (*Hordeum vulgare* L. cv. Obzor) leaves, this was noticed under severe Cu toxicity where plants were subjected to different concentrations of Cu (1.5 (control), 15, 150 or 1500 μM CuSO_4_) [59]. In *Phaseolus vulgaris*, an increase in total AsA was observed in roots when exposed to15 μM CuSO_4_ from 1 h up to one week [55], in leaves when treated with 50 μM CuSO_4_ from 24 h up to one week, [56] and in leaves when the beans were supplied with 50 μM ZnSO_4_ from 1 h up to one week [60]. Also, *Arabidopsis thaliana* exposed for 24 h showed an elevated total AsA level in the leaves when exposed to 2, 5 [26] or 10 μM CuSO_4_[17]. This elevation was also observed in *Brassica juncea* exposed to 0.007, 0.05, 5 or 10 mM ZnSO_4_ for 10 days [62] and in rice (MSE-9) exposed to 0, 10, 50 or 100 μM CuSO_4_ for one or five days [57].

In contrast, total AsA levels did not always rise in Cu- or Zn-exposed plants (Table 1). A decrease in total AsA content was observed in the roots of *Arabidopsis thaliana* plants exposed for 24 h to 2 and 5 μM CuSO_4_[26] or 10 μM CuSO_4_[17]. Similar results were reported for *Phaseolus vulgaris* roots supplied with 50 μM ZnSO_4_ up to 96 h. However, when the exposure lasted after 96 h, an increase was observed [60]. Besides Cu and Zn, Mn caused a transient increase in total AsA, followed by a decrease at high Mn exposure, which was observed in *Hordeum vulgare* seedlings exposed to 183, 1830, or 18300 μM MnCl_2_ (Table 1) [59].

## 3. Interference of Metals with Physiological Functions of AsA

### 3.1. Growth and Development

The *vtc* mutants of *Arabidopsis* and many transgenic plant species affected in AsA biosynthesis frequently show altered growth and development. To be viable, mutant plants must still contain at least a very low amount of AsA (for example: *vtc2*-mutant with 10% to 25% AsA of wild-type plants). Mutants without the metabolite AsA are lethal, which proves that it is a vital molecule for plant survival. Furthermore, there is evidence that AsA levels vary with plant developmental stages. Indeed, AsA, as well as its metabolic-related enzymes, are involved in the control of plant growth processes by controlling several basic biological processes, such as (1) the biosynthesis of hydroxyproline-rich proteins required for the progression of G1 and G2 phases of the cell, (2) the crosslinking of cell wall glycoproteins and other polymers, and (3) redox reactions at the plasmalemma involved in elongation mechanisms [30].

When wild-type plants and *vtc-1* mutants were germinated on 1% (*w*/*v*) agar containing 1/4 Hoagland’s nutrient solution, the *vtc-1* mutant showed modified shoot morphology and markedly decreased shoot biomass [67]. They also had smaller rosettes with approximately 50% of the wild-type rosette fresh weight after five weeks. Furthermore, the rosettes of the *vtc-1* mutant entered senescence earlier than those of the wildtype. The effect of the low AsA levels present in the *vtc-1* mutant could be due to the role of AsA in the plant cell cycle, or to the disruption of control mechanisms involved in cell division and/or elongation [68].

#### 3.1.1. Cell Division

Several reports showed that AsA is related with cell division in plants. Kerk *et al.*[69] reported that the amount of AsA was usually higher in the meristem than that in non-dividing cells arrested in G1 phase, such as in the maize root quiescent center. This corresponds to a high expression level of ascorbate oxidase (AO) in this cell divisioninactive tissue, which is correlated with low or undetectable levels of AsA. Thus, AsA promoted the G1 to S progression in root meristem and resulted in decreasing number of cells in the quiescent centre [69]. Further evidence for the role of AsA in controlling the transition from G1 to S phase is provided by Potters *et al.*[70]. They showed that exogenous oxidized AsA and reduced AsA have a significant impact on the cell cycle progression and thus cell division or proliferation in tobacco suspension cells [70]. In addition, *Arabidopsis vtc* mutants, which have a low content of AsA, show retarded cell division, lowered growth rate of young branches and a slow plant growth [71].

In general, most metals cause plant growth inhibition and redox imbalance. In wheat plants (*Triticum aestivum*) exposed to 1, 10 and 100 μM CdCl_2_ during 48 h, ROS were detected in the root apex. The proliferation zone of the root apical meristem was reduced in exposed plants as compared to the control. The authors suggested that Cd-induced ROS production could affect G1/S phase transition and progression through S phase [72]. Moreover, two-day-old maize seedlings exposed to 35 μM Ni(NO_3_)_2_, 10 μM Pb(NO_3_)_2_ or 3 mM Sr(NO_3_)_2_ showed a reduced mitotic index in the root cortex, determined by the increase in the cell cycle duration and accompanied by the meristem shortening. Cell division was mainly inhibited by Ni, whereas Sr and Pb affected both cell division and elongation [73]. The essential metal Zn also causes growth depression, decreased root number and length and a strong depression in root mitotic activity. This was studied in *Saccharum* supplied with 0.065 (control), 65 and 130 mg/L ZnSO_4_[74].

#### 3.1.2. Cell Wall Metabolism and Cell Expansion

Cell wall metabolism and cell growth are directly or indirectly affected by AsA, as well as by the enzyme AO [75]. Ascorbate and its oxidation products (monodehydroascorbate, MDHA; dehydroascorbate, DHA) influence plant cell expansion by a number of proposed mechanisms. One of these is the direct reaction of DHA with lysine and arginine side-chains to prevent crosslinking of cell wall proteins and polysaccharides, resulting in looser walls [76]. In addition, DHA could generate wall oxalate which might then influence free calcium levels through the formation of calcium oxalate crystals [76]. In addition, the monovalent oxidation product, MDHA, has a role in regulating cell expansion as it is generated by AO activity and increases H^+^-ATPase activity, which will then lead to increased cell expansion and solute uptake. Another mechanism is the direct scavenging of monolignol radicals involved in lignin biosynthesis by AsA, as well as the reversible inhibition of the cell wall/apoplastic peroxidases responsible for the formation of monolignol radicals [75]. In addition, there is APx sustaining cell wall plasticity by reducing the availability of H_2_O_2_ for other apoplastic peroxidase reactions and hence preventing lignification. Apoplastic peroxidases use H_2_O_2_ as oxidant to produce monolignol radicals, a reaction inhibited by AsA that will scavenge these radicals. Thus, according to Davey *et al.*[75], cell wall plasticity will be maintained due to the balance between AsA and H_2_O_2_ controlling the polymerization rate of lignin monomers. It is known that high AO activity is correlated with areas of rapid cell expansion [29].

The enzymes involved in AsA biosynthesis can also regulate cellular processes. Torabinejad *et al.*[77] reported that the enzyme VTC4 is a bifunctional enzyme that affects both myoinositol and AsA biosynthesis in plants. Myoinositol synthesis and catabolism are crucial for the production of phosphatidylinositol signaling molecules, glycerophosphoinositide membrane anchors and cell wall pectic non-cellulosic polysaccharides [77]. Another biosynthetic enzyme, GalDH, could have a role in cell expansion processes [78]. In plants with a reduced activity of GalDH, plant growth rate was decreased. The most affected plants with 80% reduction in GalDH activity showed a strong reduction in leaf and fruit size, mainly as a consequence of reduced cell expansion. All these results argue in favor of AsA and its accessory enzymes and oxidation products being closely related with the processes of cell wall metabolism and cell expansion [78].

When different plant species are exposed to Cd, cell walls of roots and leaves are directly exposed to the metal excess. In different studies, a correlation between growth reduction and increased lignin content in roots has been considered as a typical event in defense against metal stress in different plant species [79–85]. Lignification can limit the cell expansion, the capacity for nutrient uptake and thus the ability to sustain plant growth [47]. In three-day-old soybean seedlings supplied with 0, 25, 50, 75 or 100 μM CdCl_2_ for 24 h, an inhibited root growth was observed, which was followed by and associated with lignin production and related parameters. The biosynthesis of lignin involves the polymerization of monolignols primarily derived from the phenylpropanoid pathway, which commences with the deamination of phenylalanine by phenylalanine ammonia-lyase (PAL) to form cinnamate. The PAL activity increased after 50–100 μM Cd treatments, which strengthens the hypothesis that Cd induces lignification processes. Also, the cell wall-bound peroxidase activities significantly increased after 50–100 μM Cd exposure. These enzymes polymerize monolignols, which requires oxidative coupling and is dependent on H_2_O_2_. As an electron acceptor for cell wall-bound peroxidases, H_2_O_2_ plays a major role in polymerization of phenolic monomers in the lignin biosynthesis. Content of H_2_O_2_ was increased when soybean seeds were exposed to 50–100 μM Cd, and the production of H_2_O_2_ has been correlated with the stiffening of cell walls as growth ceased and cells differentiated. Furthermore, the production of lignin was elevated, which stiffens the cell wall and restricts plant growth [84]. In the roots of 21-day-old *Matricaria chamomilla* plants exposed to 60 or 120 μM CdCl_2_ for seven days, the activity of PAL was stimulated and accompanied with an increased content of lignin [83]. Also in five-day-old soybean seedlings treated with 0.2–1 mM CdCl_2_, the lignin content significantly increased in the root tips. Moreover, the lignin biosynthesis related enzymes, peroxidases and laccases were enhanced during Cd treatment [82]. Tamás *et al.*[85] showed in barley seeds (*Hordeum vulgare*) exposed to 1 mM CdCl_2_ that stress activated several H_2_O_2_ generating enzymes, e.g., NADPH-oxidase, which probably contributes to general stress induced morphological changes of barley root tips, such as root thickening, cell wall modifications and lignification. These responses were accompanied by root growth inhibition due to the enhanced rigidification of cell wall and accelerated differentiation of root cells [85]. A high concentration of Cu also induced high accumulation of lignin in the roots of 21-day-old *Matricaria chamomilla* plants containing 60 or 120 μM CuCl_2_ for seven days [83]. This is in accordance with the observation in soybean exposed to 1–10 mM CuSO_4_ for 1–72 h, in which the lignin contents were significantly increased in the roots after 24 h [81]. The aim of this early synthesis could be the immobilization of toxic metals in negatively charged sites of cell walls and restriction of their apoplastic transport [81]. In a study of Chaoui *et al.*[80], seeds of pea (*Pisum sativum*) were treated with 20 and 100 μM Cd(NO_3_)_2_ or 20 and 100 μM CuSO_4_ for four days. The activities of lignifying peroxidases were not significantly altered, and NADH oxidase activity was even inhibited during Cu treatment. In comparison to Cu, exposure to Cd stimulated the cell wall-lignifying peroxidases and increased the activity of NADH oxidases. Microsomal APx activity, which was very low in the control, was markedly enhanced in metal-exposed plants. It is known that membrane-associated peroxidases are able to oxidize AsA and, consequently, could have an antioxidant role that seems to be stimulated in roots of Cd- and Cu-treated pea. This is in addition to their contribution to the stimulation of the lignification process [80]. In another study, the effect on guaiacol and syringaldazine peroxidases were investigated in roots and primary leaves of 11-day-old *Phaseolus vulgaris* seedlings exposed to 15 and 50 μM CuSO_4_ and 50 μM ZnSO_4_[79]. After exposure to 15 μM Cu, the capacity of guaiacol peroxidase increased from 24 h onward and the syringaldazine peroxidase activity rose significantly at 48 h in the roots of *Phaseolus vulgaris*. In contrast to Cu, a limited effect of Zn on the enzyme capacities was observed in the roots. In the primary leaves, it was observed that exposure to Cu or Zn resulted in increased capacities of both peroxidases. The function of these peroxidases in lignin biosynthesis is receiving more attention since many of the peroxidases studied are localized in the apoplast and play a key role in cell wall lignification. It is established that cell wall-associated peroxidases catalyze the final enzymatic step in lignin biosynthesis, *i.e.*, the oxidation of cinnamyl alcohols [79].

#### 3.1.3. Senescence

Senescence implies the coordinated degradation of macromolecules and the mobilization of regained nutrients such as nitrogen, carbon and minerals from senescing tissues to developing parts of the plant [86]. It is also characterized by a series of physiological and biochemical changes, such as chlorophyll degradation and a declining photosynthetic activity due to decreased expression of the Rubisco small subunit and chlorophyll a/b binding proteins. These genes are termed senescence-down-regulated genes (SDGs), while other genes are upregulated and therefore called senescence-associated genes (SAGs) [86,87]. In the later stages of senescence, cell peroxidation and DNA degradation occurs, which results in disintegrated organelles [87]. It is well known that senescence is related to an increased level of free radicals, especially those derived from oxygen, as well as to a loss of antioxidant activity [86].

Barth *et al.*[87] showed that AsA influences senescence by modulating the expression of SAGs. The leaves of *vtc1* mutant plants lost chlorophyll more quickly and entered senescence earlier than the wild-type leaves. In addition, an upregulation of the expression of selected SAG transcripts was observed in the mutants, suggesting that AsA deficiency induces a senescent phenotype [88]. Due to its essential function as cofactor for enzymes involved in the biosynthesis of gibberellins, abscisic acid and ethylene, AsA together with various phytohormones has a role in the senescence process. Abscisic acid and ethylene promote senescence, in contrast with gibberellic acid, which prevents senescence [88]. Thus, the redox status of AsA may play a role in senescence by influencing complex phytohormone-mediated signaling networks, by modulating ROS accumulation and by stimulating the expression of SAGs.

Exposure to Cd is suggested to induce or accelerate leaf senescence. In senescent pea leaves, a role for the peroxisomal protease in the metabolic transition of leaf peroxisomes into glyoxysomes has been elucidated by Distefano *et al.*[89]. Pea (*Pisum sativum*) plants exposed to 50 μM CdCl_2_ for 28 days were studied to determine the effect of Cd on the peroxisomal metabolism whether these organelles are representative of the overall senescence symptoms promoted by Cd in leaves. The results showed that the peroxisome metabolism and proteolytic activity provide evidence for a Cd-induced senescence in pea leaves and suggest a role for peroxisomal proteases in the metabolic changes induced by metal stress [90]. Furthermore, the Cd-induced changes observed by electron microscopy in the chloroplast structure showed the same pattern as that observed in other plant species treated with Cd, and which are very similar to those found in senescent tissues. Taken together, these results indicate that Cd induces senescence symptoms in leaf peroxisomes [90]. Also, in tomato (*Lycopersicon esculentum*. Mill. cv. 63/5F1) treated with 50 μM CdCl_2_ for one week, it was shown that Cd induces peroxisomal senescence in leaves by activating the glyoxylate-cycle enzymes, malate synthase and isocitrate lyase, as well as peroxisomal peptidases, the latter being a well-known leaf senescence-associated factor [91].

### 3.2. Photosynthesis

Ascorbate is present in the cytosol, chloroplasts, vacuoles, mitochondria and cell wall. Because of its central role in photosynthesis, the AsA concentration in chloroplasts can be as high as 50 mM as observed for spinach [76]. Firstly, AsA protects the photosynthetic apparatus against ROS that are formed by oxygenic photoreduction in photosystem I (PSI) (Mehler reaction) [36]. As CAT is not present in chloroplasts, H_2_O_2_ is reduced by APx using AsA as reducing agent [92]. Secondly, AsA can directly scavenge superoxide (O_2_°^−^), hydroxyl radicals (°OH) and singlet oxygen (^1^O_2_) [36]. Thirdly, AsA contributes to the regeneration of α-tocopherol from α-tocopherol radicals that are formed during the reduction of lipid peroxyl radicals, thus protecting chloroplast membranes against oxidative degradation [36]. Furthermore, MDHA, the primary oxidation product of AsA, can act as a direct electron acceptor to PSI [92]. Finally, AsA is a co-factor for violaxanthin de-epoxidase (VDE), which is involved in photoprotection mediated by the xanthophyll cycle (Figure 1) [76,93]. The vital role of AsA in photosynthesis is demonstrated by transgenic rice plants with suppressed expression of GalLDH. This resulted in a loss of chlorophyll, a lower Rubisco protein content and a lower rate of CO_2_ assimilation, leading to a slower plant growth rate and lower seed production [94].

Toxic metals generally influence the functions of the photosynthetic apparatus. They may interact with the photosynthetic apparatus at various levels of organization and architecture, such as in leaf tissues like stomata, mesophyll and bundle sheath cells or interaction of metals with cytosolic enzymes [95]. Metals may damage the electron transport activity by the induction of peroxidation and loss of thylakoid membrane integrity, thus altering the function of PSI and PSII [95,96]. The altered chloroplast structure and the substitution of Mn by Zn or Cd leads to the inactivation of the oxygen evolving complex and hence this has consequences for the electron donation to PSII [96]. Finally, there is evidence that Cd has an influence on the activity of the chloroplasts by modifying chlorophyll content, which has been attributed to reduced chlorophyll synthesis or to enhanced enzymatic degradation [49,97–100]. Ding *et al.*[97] showed a significant loss of chlorophyll and carotenoid content in the leaves of *Alternanthera philoxeroides* exposed to 0.5, 1, 2, 5 and 10 μM CdCl_2_ for 48 h, which further diminished with increasing Cd concentrations [97]. Also, in seeds of mustard (*Brassica campestris*) exposed to 0–100 mg/kg CdCl_2_, the chlorophyll content was significantly decreased with increasing Cd concentration in the soil [98]. In another study, maize cultivars 32D99 and 3223, respectively tolerant and sensitive to Cd stress, were treated for eight days to 0.3, 0.6 and 0.9 mM Cd(NO_3_)_2_. Also, in this study increasing Cd concentrations inhibited chlorophyll biosynthesis; the highest carotenoid content was also observed in control plants for both cultivars, which further diminished with increasing Cd concentrations. Furthermore, it was shown that Cd inhibited the photoactivation of PSII. A decline in parameter F_M_, the maximum fluorescence in the dark-adapted state obtained by a saturation pulse (white light), suggests a change in the ultrastructure of thylakoid membrane, affecting the electron transport rate. The ratio of F_V_/F_M_, the quantum efficiency of PSII open centers in dark-adapted seedlings, is often used as a stress indicator and describes the potential yield of the photochemical reaction and is decreased when exposed to increasing Cd concentrations for both cultivars [99]. Also in *Oryza sativa*, after 12 days of exposure to 50 μM CdCl_2_, the F_V_/F_M_ of Cd-sensitive mutant leaves was significant lower than in the wildtypes. After 3 days of restoration, F_V_/F_M_ of the wild-type leaves was increased near the initial value, while F_V_/F_M_ of the mutant was still significantly lower than that of the controls. The content of chlorophyll and carotenoid was decreased after exposure to Cd and the contents were lower in the leaves of the mutants than in the wild-types [100]. In *Brassica juncea* grown for seven days in 0, 50 or 200 μM Cd(NO_3_)_2_, Cd negatively affected chlorophyll and carotenoid contents and activated the xantophyll cycle. The decreased β-carotene content observed in Cd-exposed leaves may therefore allow ROS accumulation in the photosynthetic apparatus, in its turn leading to oxidative degradation of chlorophylls, destabilizing the structure of PSII. A significant increase in the de-epoxidation index of *Brassica juncea* induced upon Cd exposure suggests the need to protect the photosynthetic apparatus of Cd-exposed plants from photoinhibition [49].

In general, the effect of metal influence on plants is largely a strong and fast inhibition of growth processes of the above- and underground parts, as well as the activity decrease of the photosynthetic apparatus, often correlated with progressing senescence processes [24]. It is also evident that AsA plays an important role in these functions; however, further research is needed to explore the interaction between metals, the physiological functions and AsA.

## 4. Metabolism of AsA

A number of products are metabolized from AsA, including l-tartrate and oxalate. The accumulation of tartrate is restricted to a handful of plants, while oxalate is widely distributed and appears predominantly as crystals of calcium (Ca) oxalate [101]. Calcium oxalate synthesis in plant tissues could therefore be involved in the regulation of cellular Ca concentration [29,101]. The extent of accumulation of tartrate in plants remains unclear. It is known that the dominant organic acid in grape (*Vitis vinifera*) berries is tartrate. Berry tartrate is largely responsible for controlling the pH of the juice in winemaking. Addition of tartrate during vinification minimizes oxidative and microbial spoilage [102].

The details of tartrate and oxalate formation vary between species. It became apparent that the AsA cleavage pathways showed species-specific differences that remain unresolved. Whereas oxalate may be derived from photorespiratory intermediates such as glycolate [29,101], the main source of oxalate in various species is AsA [39]. To form oxalate, the AsA carbon skeleton is cleaved between the C2/C3 position and is formed with the carbon atoms 1 and 2. For some other plant species, the carbon atoms 3 to 6 form l-threonic acid, which may be further oxidized to form tartrate. In the grape family, the formation of tartrate is performed by the C4/C5 cleavage [101,103].

The oxalate crystals can be used as a sequestration mechanism of toxic metals. The immobilization of excess Mn in these crystals, reported in earlier studies [104,105], is supposed to be a detoxification mechanism. In the leaves of *Phaseolus vulgaris* (tolerant and susceptible genotypes to Mn stress) receiving 200 μM MnSO_4_, a depletion of AsA was observed without any changes in its redox state that was even more pronounced in the susceptible genotypes [106]. Also another study reported that AsA levels were diminished without increasing the percentage of oxidized forms of AsA under the highest concentration of Mn exposure [59]. During this research, they used five-day-old *Hordeum vulgare* seedlings that were treated with 183, 1830, 18,300 μM MnCl_2_ over five days. The AsA depletion was suggested in both studies to be due to an elevated synthesis of oxalate, at the expense of AsA, needed for the immobilization of Mn in oxalate crystals.

The use of oxalate as a detoxification mechanism has also been reported for other toxic metals. It was suggested that excess chromium (Cr) in rice plants is bound to undissolved or low-bioavailable compounds such as oxalate [107] when two rice genotypes (*Oryza sativa* L. cv. Xiushui 113 and cv. Dan K5) were exposed to different amounts of K_2_Cr_2_O_7_ (0, 10, 50 and 100 μM) during 10 days. In *Leersia hexandra*, treated with 0, 5, 30 and 60 mg/L CrCl_3_, Cr was bound to oxalate in the leaves [108]. Also, in the leaves of *Eichhornia crassipes* exposed to Cr, this might be bound to oxalate ligands, while in the roots Cr was hydrated by water [109]. These results indicate that oxalate can be important in Cr accumulation and detoxification.

## 5. Localization of AsA and its Biochemical Reactions

### 5.1. Subcellular Localization of AsA

Although mitochondria are the only compartments in *Arabidopsis* in which the synthesis of AsA molecules can be completed, the highest concentration of AsA in non-stressed wild-type plants was detected in nuclei and the cytosol [110]. This demonstrates that AsA must be transported via the cytosol into other cellular compartments. Ascorbate has an important function in the detoxification of ROS produced in chloroplasts under non-stress conditions, but especially under high light stress when the amount of AsA reaches similar levels as those observed in the cytosol [110]. During high light conditions, increased levels of AsA were also observed in the vacuoles [110]. H_2_O_2_ that diffuses into the vacuoles is detoxified, while AsA is oxidized to MDHA and DHA which are then transported into the cytosol for reduction to AsA [111]. The high levels of AsA in the nuclei, which are approximately similar to those in the cytosol, may be due to the nucleus being freely permeable to AsA. The possible roles of AsA in the nucleus of plant cells is still unclear [110], but it could be essential for the protection of DNA against oxidative modifications as demonstrated for mammalian cells [112]. In addition, up to 10% of the AsA content of the whole leaf is localized in the apoplast. A key function of this apoplastic AsA is redox buffering, which protects the plasma membrane from oxidative damage [113].

### 5.2. Role of AsA as a Cellular Antioxidant

One of the major roles of AsA is being an antioxidant that protects metabolic processes against H_2_O_2_ and ROS [114]. Ascorbate protects the plant from oxidative stress, and when taken up by the diet (vitamin C) it can also protect mammals from various chronic diseases that would otherwise result from oxidative stress [30]. In plants, ROS are generated during aerobic cellular metabolism and photosynthesis, as well as by biotic and abiotic stresses such as drought, ultraviolet, wounding, ozone and a range of pollutants [30,115]. Ascorbate can directly react non-enzymatically with O_2_°^−^, H_2_O_2_ and ^1^O_2_, generating MDHA and DHA [28,29]. In addition, it can act as a secondary antioxidant by regenerating α-tocopherol or in the regeneration of zeaxanthin in the xanthophyll cycle [114]. As a cofactor, AsA influences many enzyme activities through a synergistic cooperation [116–118]. One such important enzyme, APx, is essential for the detoxification/reduction of H_2_O_2_ to water and has a high specificity and affinity for AsA, which is used as electron donor for this reduction. As such, APx is connected to and forms an important component of the AsA-GSH cycle (Figure 1). The outcome of this cycle is that H_2_O_2_ is reduced to water by the reducing power derived from NADPH. In a first step, H_2_O_2_ is reduced by APx using AsA that is oxidized to MDHA, which in turn is transferred back into AsA via the activity of monodehydroascorbate reductase (MDHAR) using NADPH. When MDHA is not immediately reduced, it disproportionates spontaneously to DHA and then to AsA. DHA is reduced to AsA via the action of dehydroascorbate reductase (DHAR), with glutathione (GSH) as an electron donor being converted to glutathiondisulfide (GSSG). Finally, GSSG is reduced with electrons from NADPH in a reaction catalyzed by glutathione reductase (GR) [29,67,119,120]. Thus, cellular levels of reduced AsA that are able to function in ROS scavenging are determined by both, the rate of AsA synthesis and the rate of recycling reduced AsA from its oxidized forms via DHAR and MDHAR.

Recent evidence suggests that AsA plays a prominent role in protection of plants against several environmental stresses. Dehghan *et al.*[121] showed that exogenous application of AsA via a pretreatment resulted in an improved germination percentage and growth of salt-stressed seedlings of soybean cultivars. They suggested that AsA can protect soybean seedlings from salt-induced oxidative stress through an increase in their antioxidative capacity [121]. Also for several other stressors, such as biotic stress [122] and UB-V [123], a protective function of AsA has been suggested. Here, we focus on studies suggesting a role for AsA in plants exposed to excess metals.

#### 5.2.1. Protection of AsA against Excess of Non-Essential Metals

Cadmium is a non-essential non-redox active metal and the effects of this metal on the AsA-GSH cycle have been examined in several studies (Table 1). During short-term (24 h) exposure of *Arabidopsis thaliana* plants to 10 μM CdSO_4_, an elevated level of reduced AsA was reported in roots [17] and in leaves [45]. In contrast, a longer exposure time (>24 h) to Cd resulted in a decrease in the content of reduced AsA in *Ceratophyllum demersum* treated with 10 μM CdCl_2_ during one week [48]. This kinetic trend of an increase followed by a decrease was also found in *Pinus sylvestris* treated with 0, 5 and 50 μM CdSO_4_ during 6–96 h [47]. The initial increase in the amount of reduced AsA in Cd-exposed roots was followed by a decrease or even a drop below the detection limit after 24 h of Cd stress. Furthermore, decreases in the redox state DHA/AsA ratio were observed in *Arabidopsis thaliana* exposed to 10 μM CdSO_4_ during 24 h in roots [17] and leaves [45], while increased DHA level and DHA/AsA ratios were reported in different plant species exposed to Cd stress for longer times (>24 h). These latter observations were shown in *Bechmeria nivea* Gaud exposed to 0, 1, 3 and 7 mg/L Cd during 1–10 days [50], in leaves of *Phaseolus vulgaris* treated with 2 μM CdSO_4_ during 0–72 h [46] and in *Ceratophyllum demersum* supplied with 10 μM CdSO_4_ during 1 week [48]. These results indicate that initially stimulation of AsA biosynthesis occurs increasing the level of reduced AsA that can act as a primary antioxidant. Thereafter, AsA can concomitantly function as a cofactor of APx in the AsA-GSH cycle to enzymatically detoxify ROS. Together, usage of AsA as a direct antioxidant and as a cofactor for APx goes hand in hand with an enhanced AsA oxidation in Cd stress. Indeed the activities of APx and/or GR were generally elevated in different plant species exposed to various concentrations of Cd for short (<24 h) and long (>24 h) periods (Table 1). Interestingly, in *Pinus sylvestris* exposed to 0, 5 and 50 μM CdSO_4_ during 6–96 h, APx activities were initially decreased after 6h of exposure to 50 μM Cd, but had recovered and were significantly increased after 24 h, which led to an accumulation of H_2_O_2_ after 6 h that was less pronounced after 24 h [47]. This transient rise in H_2_O_2_ levels may be important for it to act as a signaling molecule in the activation of cellular defenses, including APx [124,125]. In the same study, the redox state of AsA was also initially more oxidized in the presence of 50 μM Cd, as AsA was removing H_2_O_2_ non-enzymatically, and enhanced activities of MDHAR were reported [47]. In contrast, in *Ceratophyllum demersum* plants treated with 10 μM CdSO_4_ during one week, reduction in the activities of MDHAR and DHAR were observed [48]. It has to be kept in mind that experimental set-up, *i.e.*, Cd exposure concentrations and duration, as well as the plant species and their inherent uptake mechanisms, metal homeostasis and metal tolerance are important determinants in the outcome of Cd-induced oxidative stress responses. Therefore, generalization over different species and studies is hard to perform.

Aluminum is a non-essential redox active metal, which also decreases reduced AsA levels together with increased DHA/AsA ratios in roots and shoots of a rice cultivar of India Pant-12 exposed to 80 μM or 160 μM Al_2_(SO_4_)_3_ during 0 to 20 days (Table 1) [52]. Nevertheless, plants tried to counteract this decline in reduced AsA by enhancing MDHAR and DHAR activities. In Al-exposed rice seedlings, these stimulations were more pronounced in the presence of 80 μM Al as compared to 160 μM Al. Furthermore, an increased H_2_O_2_ content was observed in these rice plants exposed to 160 μM Al, while a decline was shown at 80 μM Al exposure. The authors suggested that the generation of severe oxidative stress in rice plants due to Al treatment of 160 μM is responsible for the failing of the antioxidative defense system. The observed elevation in APx activity in rice seedlings may have been sufficient to remove H_2_O_2_ at 80 μM exposure, but the increased APx activity under 160 μM Al exposure may not have been sufficient, hence H_2_O_2_ levels increased [52].

The regeneration of AsA was also studied in *Nicotiana tabacum* wild-type SR-1 plants and in transgenic tobacco plants overexpressing *Arabidopsis thaliana* cytosolic DHAR (DHAR-OX) and MDHAR (MDHAR-OX) exposed to 400 μM AlCl_3_ (Table 1) [53]. Under Al exposure, the DHAR-OX plants showed higher levels of reduced AsA and lower DHA contents and thus a lower DHA/AsA ratio than that in wild-type and MDHAR-OX plants. This indicates an increased efficiency of AsA regeneration by overexpressing DHAR, but not by overexpressing MDHAR. Furthermore, there was a higher increase in APx activity in DHAR-OX plants under Al treatment as compared to wild-types. These findings suggest that overexpression of DHAR can stimulate the reduction of AsA under Al treatment and as such contributes to Al tolerance in tobacco.

#### 5.2.2. Protection of AsA against Excess of Essential Metals

Copper is an essential redox-active metal, and, in excess, influences the content of AsA in the cell (Table 1). Similar to non-essential metals, Cu induces an increased DHA/AsA ratio in different plant species exposed to various concentrations of CuSO_4_. This result was seen, for example, in a study of Tewari *et al.*[58], where mulberry (*Morus alba* L. cv. Kanva-2) plants were exposed for 25 or 50 days to 1 μM Cu used as control condition and up to 100 μM Cu (excess supply). Furthermore, other studies showed an increase in DHA/AsA; in the roots of *Arabidopsis thaliana* seedlings exposed to 2 and 5 μM CuSO_4_ during 24 h [26] and in roots of *Phaseolus vulgaris* treated with 15 μM CuSO_4_ during 1 h up to 168 h [55]. Roots are in direct contact with the nutrient solution and, hence, with the applied Cu. Moreover, Cu is mainly retained in the roots [26] and as Cu easily performs monovalent oxidations, it can directly react with reduced AsA and hence stimulate its oxidation.

In contrast, in the leaves of *Phaseolus vulgaris* exposed to 50 μM CuSO_4_ during 0 h up to 168 h, a decline of the DHA/AsA ratio was shown. Here, reduced AsA was always elevated and a decrease in DHA was shown after 48 h [56]. Also in the leaves of *Arabidopsis thaliana*, an increased AsA level was observed after 24 h treatment of 10 μM CuSO_4_, which was in contrast with the roots of *Arabidopsis* with the same treatment [17] (Table 1). In addition, an enhancement of the activities of APx and/or GR was reported in different plant species treated with various concentrations of Cu for short- (<24 h) and long-term (>24 h) exposures (Table 1). Furthermore, the activities of MDHAR and DHAR were increased in roots [55] and leaves [56] of *Phaseolus vulgaris* exposed to, respectively, 15 μM and 50 μM CuSO_4_, which resulted in an elevated AsA content for both plant organs, but in an increase of DHA in the roots and a decrease of DHA in the leaves. Elevated activities of APx and GR, together with the alterations of the DHA/AsA ratio suggest an activation of AsA–GSH cycle that works efficiently for the leaves, but is insufficient to maintain the reducing potential of the system as indicated by the increased DHA/AsA ratios in the roots.

Zinc and Ni are both also essential metals, but in contrast to Cu, they are not redox active. An increased DHA/AsA ratio was observed in the roots and leaves of *Phaseolus vulgaris* after exposure to 50 μM ZnSO_4_ during 1 h up to 168 h (Table 1) [60]. In roots, already after 5 h the DHA/AsA was elevated due to the temporary decrease of reduced AsA. In the leaves, there was also an enhancement of the DHA/AsA ratio, but after longer exposure to Zn (96 h). In the roots of *Phaseolus vulgaris*, elevations in the contents of reduced AsA and DHA were observed after 96 h and 72 h of Zn treatment, respectively. The enhancement of DHA content was greater than the enhancement of reduced AsA, resulting in a higher DHA/AsA ratio. During germination (six days) of pigeon pea seedlings (LRG30-long duration type and ICPL87-short duration type) supplied with 2.5, 5 and 7.5 mM Zn, a decrease of reduced AsA was shown, which is corresponding to the initial decrease in reduced AsA in the roots of *Phaseolus vulgaris* exposed to 50 μM ZnSO_4_[61]. In both, leaves and roots of *Phaseolus vulgaris* exposed to 50 μM Zn [60] and in *Brassica juncea* exposed to 5 and 10 mM ZnSO_4_ during 10 days [62], the APx activity was enhanced. Elevated activity of GR was observed in pigeon pea seedlings supplied with 2.5, 5 and 7.5 mM Zn during six days [61], in *Brassica juncea* exposed to 5 and 10 mM ZnSO_4_ during 10 days [62] and in the leaves of *Phaseolus vulgaris* treated with 50 μM ZnSO_4_ during 1 h up to 168 h, suggesting that the AsA–GSH cycle is activated but nevertheless insufficient to maintain the redox state.

The DHA/AsA ratio was also higher with increasing Ni concentration in rice plants (*Oryza sativa* L.) exposed to 200 μM and 400 μM NiSO_4_ up to 20 days (Table 1) [63]. Both, the amounts of AsA and DHA were higher in rice plants treated with Ni. This does not correspond with the results of Rao and Sresty [61], in which AsA content decreased in pigeon pea seedlings (LRG30-long duration type and ICPL87-short duration type) supplied with 0.5, 1 and 1.5 mM Ni during six days of germination. Also the effect of excess Mn on AsA redox cycling has been examined (Table 1). A decline in reduced AsA was shown in Mn-sensitive cowpea (*Vigna unguiculata*) cv. TVu 91 exposed to 50 and 100 μM MnSO_4_ during 0, 10 and 20 days [64] and in *Oryza sativa* L. plants exposed to 3 and 6 mM MnCl_2_ during six days [65]. In addition, the DHA/AsA ratio was increased in *Oryza sativa* L. plants exposed to 3 and 6 mM MnCl_2_ during six days [65]. In these plants treated with Mn, an increased activity in MDHAR, DHAR and GR was observed. These stimulated activities are supposed to induce AsA regeneration to maintain their requisite levels to inhibit the damage by Mn exposure. However, a decline in reduced AsA was shown, suggesting that enhanced activities of these enzymes were not sufficiently recovering reduced AsA from its oxidized forms [65]. In contrast, Mn-tolerant cowpea cv. TVu 1987 exposed to 50 and 100 μM MnSO_4_ showed under high Mn treatment an enhancement in the activities of AsA-regenerating enzymes like MDHAR and GR, and in these plants, no elevations of DHA were shown. This suggests that, in the Mn-tolerant genotype, the induction of enzymatic activity was sufficient, as opposed to the sensitive genotype.

### 5.3. The Interaction of AsA with the Antioxidant α-Tocopherol

In an indirect way, AsA is involved in ROS scavenging by regenerating α-tocopherol or by its role in zeaxanthin synthesis in the xanthophyll cycle (see section 3.2) [114]. Alpha-tocopherol is the major vitamin E compound found in the membranes of the chloroplast envelope, thylakoid membranes and plastoglobuli [126]. It is a lipophilic antioxidant that interacts with the polyunsaturated acyl groups of lipids to stabilize membranes, but is also able to quench various ROS and oxidized lipids [114]. Tocopherols predominantly protect polyunsaturated fatty acids (PUFA) from being oxidized [114] by preventing the propagation of lipid peroxidation, either by scavenging photosynthesis-derived ROS (mainly O_2_°^−^ and OH°) or lipid peroxyl radicals in thylakoid membranes [126]. While scavenging lipid peroxyl radicals, α-tocopherol itself is oxidized and can be reduced by reacting with AsA and other antioxidants [114]. In general increased α-tocopherol levels contribute to plant stress tolerance. When 18-month-old rosemary (*Rosmarinus officinalis*), sage (*Salvia officinalis*) and lemon balm (*Melissa officinalis*) were exposed to drought stress, α-tocopherol levels increased in the three species studied [127]. Exogenous AsA administration partly prevented α-tocopherol oxidation in osmotocially shocked chloroplasts in the light. Furthermore, chloroplastic α-tocopherol and AsA levels increased and no photo-inhibitory damage could be observed at relative leaf water contents between 58% and 86% in any of the species studied. These results suggest that a positive interplay exists between both AsA and α-tocopherol, where AsA may indirectly protect α-tocopherol by scavenging ROS and may participate in the recycling of α-tocopherol radicals [127]. In soybean seeds supplemented with 500 μM Fe-EDTA for 2 to 6 h, the content of α-tocopherol was not affected in the embryonic axes. However, after 24 h, significant increases in α-tocopherol concentration were observed during the initial steps of imbibition [128]. Also in two-week-old *Arabidopsis thaliana* plants exposed to either 5 or 50 μM CdCl_2_, a significantly increased α-tocopherol content was able to prevent Cd-induced oxidative damage in chloroplasts [126]. Furthermore, transgenic *Brassica juncea* plants overexpressing the γ-TMT (γ-tocopherol methyl transferase) gene had a six-fold increase in the level of α-tocopherol in comparison to the wild-type plants, and were used to test if the increased α-tocopherol content would confer advantage to the plants exposed to Cd stress [129]. They observed that the percentage germination of transgenic *Brassica juncea* seeds on medium supplemented with 20 mM CdCl_2_ for 72 h was much higher (35.2%) than the germination of wild-type seeds under the same conditions (5.9%). Thus, the transgenic *Brassica juncea* plants had enhanced tolerance to the induced Cd stress, which was reflected in the photosynthetic performance [129]. These studies showed that α-tocopherol is increased and needed when plants are exposed to Cd stress. Further research into the link between α-tocopherol and AsA under metal stress is needed and deserves special attention.

### 5.4. AsA Oxidation by APx or AO Serves Differential Functional Goals in Plant Cells

Ascorbate peroxidase is an important AsA oxidizing enzyme using AsA as a reductant in the control of H_2_O_2_ levels in plant cells. Different APx isoforms exist in various cell compartments differentially responsive to the redox status, namely the cytosol, chloroplasts, mitochondria, peroxisomes and glyoxysomes [39]. Hydrogen peroxide can be formed by a two-electron reduction of O_2_ at the level of the chloroplast and mitochondrial electron transport chains. Oxygen reduction by PSI and removal of the resulting H_2_O_2_ by APx contributes to the regulation of the redox state of photosynthetic electron carriers [39]. A study of Karpinski *et al.*[130] showed that in four-week-old *Arabidopsis thaliana* exposed to a white light pulse, the transcripts of isoforms APx1 and APx2 (cytosolic isoforms) was rapidly and strongly increased.

Metals interfere with plant electron transport chains by enhancing electron leakage [131]. These electrons can directly reduce O_2_ leading to H_2_O_2_ production and many studies report increased APx activities as a result. Elevated APx activities were observed in Cd-exposed *Ceratophyllum demersum*[48], *Phaseolus vulgaris*[46], *Bechmeria nivea* gaud [50] and *Arabidopsis thaliana*[17,26]. Furthermore, rice seedlings exposed to 160 μM Al_2_(SO_4_)_3_ showed an increased H_2_O_2_ content, while a decline was shown during 80 μM Al exposure [52]. This suggests that plants exposed to the highest Al concentration suffer from severe oxidative stress and are unable to counteract through their antioxidative defense system. Nonetheless, APx activity increased after exposure to both concentrations [52]. Tobacco plants overexpressing the *Arabidopsis thaliana* cytosolic DHAR showed an increased APx activity after 24 h exposure to 400 μM AlCl_3_[53]. In addition, excess levels of the essential metals Cu [26,55–58], Zn [60,62], Ni [63] and Mn [65], resulted in enhanced APx activities.

Another enzyme catalyzing AsA oxidation is AO, which is mainly expressed in cell walls of fast growing plant cells [29]. Although several studies unraveled biological functions for AO, its exact role still remains to be elucidated. Nonetheless, AO has been implicated as an apoplastically AsA oxidizer during cell elongation [132,133]. In Tobacco BY-2 cells, DHA accumulation occurred after four days in the elongation culture, potentially caused by apoplastic AO [132]. Pignocchi *et al.*[133] showed that enhanced AO activity positively affects plant growth with regard to both height and biomass. They also demonstrated that changes in apoplastic AO activity strongly affect the oxidation of apoplastic AsA contents without significant changes in whole leaf AsA content. While elevated AO activity leads to increased oxidation of the apoplastic AsA pool, decreased AO activity enhanced the amount of AsA as compared to DHA [133]. The apoplastic redox state—regulated by AO activity—modulates plant growth and defense responses by regulating signal transduction cascades and gene expression patterns [134]. It was also suggested that metals can influence the redox state of the apoplast potentially by interfering with AO activity, which certainly deserves further investigation. Furthermore, Noctor and Foyer [116] and Fotopoulus *et al.*[135] reported that AO could be a key regulator of the extracellular redox state and is able to alter the expression and activities of several AsA-related enzymes. There is clearly a great need to explore the role of the apoplastic AsA pool and AO even more, and not only in fast-growing cells but also in differentiating and lignifying tissues.

### 5.5. Other Biochemical Reactions

Ascorbate is an important cofactor of vital enzymes, thereby facilitating their catalyzed reactions. More specifically, it interacts with mono- or dioxygenases that contain Fe or Cu at their active site and require AsA to acquire maximal activity. The function of AsA is to maintain these transition metal ion centers in a reduced form [75]. In addition, VDE also requires AsA to function in the photoprotective xanthophyll cycle (see Section 3.2) [30]. This cycle involves the conversion of violaxanthin to zeaxanthin in light-exposed leaves. Zeaxanthin is involved in non-photochemical quenching by dissipating excitation energy as heat [36]. Moreover, AsA acts as a prosthetic group for prolyl and lysyl hydroxylases, catalyzing the synthesis of hydroxyproline and hydroxylysine, respectively [30]. Finally, AsA is shown to be a cofactor for different enzymes involved in the synthesis of ethylene, gibberellins and anthocyanins [30].

As demonstrated in several studies (Table 1), metal stress affects plant AsA contents. A change in the AsA redox can also have an impact on the activities of enzymes that require AsA as cofactor. Nonetheless, further research is needed to explore the connection between metal stress, AsA and its involvement in different biochemical reactions as described above.

## 6. Conclusions

In general, AsA is present in all subcellular compartments and functions in many physiological processes of plants growing under non-stress conditions. When plants are exposed to excess metals, they generate excessive amounts of ROS leading to oxidative stress, which affects the biosynthesis of AsA and/or the functioning of the AsA-GSH cycle. General similarities involving the responses of AsA to metal stress can be observed. During short term (±24 h) plant exposure to Cd or Al, an enhancement of the reduced AsA is reported, which coincides with a decrease in DHA/AsA. On the contrary, a decline of reduced AsA and/or an increase in DHA was shown during long term exposures along with an elevated ratio of DHA/AsA. With increasing time exposure and dose concentrations, this results in a shift to the oxidative side along with a decrease in efficiency of the antioxidative ability. A general trend towards an increased DHA/AsA ratio was also seen when plants were exposed to essential elements such as Cu and Zn. However, a distinction should be made between leaf and root responses. Whereas roots mainly demonstrated a decrease in reduced AsA, together with an increase in the ratio DHA/AsA, an increase of AsA was observed in leaves. The fact that the AsA pool in leaves remains reduced suggests an efficient use of the AsA–GSH cycle, suggesting that signal molecules might be involved in the induction of this defense system against oxidative stress as a result of root metal uptake and/or translocation.

Exposure to metals negatively affects the AsA–GSH cycle causing a more oxidized DHA/AsA redox state. It is clear that this potentially influences the role of AsA in the processes discussed in this review such as cell division, cell wall biosynthesis and cell differentiation, senescence and its key protective role of neutralizing ROS formed during photosynthetic and respiratory reactions in chloroplasts and mitochondria, respectively. The fact that excess metals interfere with the physiological processes in which AsA is involved, makes a direct link plausible. Future research in the effects of metals may establish more direct correlations between AsA, metals, and physiological alterations or dysfunctioning under metal stress.

## Figures and Tables

**Table 1:** Exposure to excess metals has consequences for the biosynthesis of AsA and the antioxidant properties expressed as (1) the amount of reduced and oxidized ascorbate, *i.e.*, AsA and DHA and (2) the AsA-GSH cycle activities. The effects of excess Cd, Al, As, Pb, Cu, Zn, Ni and Mn are shown along with the experimental upset and the use of plant material.

	Plant	Condition	Biosynthesis/content of total AsA	Antioxidant		Ref.
				Ratios	AsA-GSH cycle	
**NON-ESSENTIAL ELEMENTS**						
**Cadmium (Cd)**	*Arabidopsis thaliana*	0, 5, 10 μM CdSO_4_ 24 h	↑ Total AsA (roots)		↑ APX, GR (leaves)	[26]
	*Arabidopsis thaliana*	0, 5, 10 μM CdSO_4_ 24 h	↑ Total AsA (leaves)	↑ Reduced AsA (leaves) ↓ DHA/AsA (leaves)		[45]
	*Arabidopsis thaliana*	10 μM CdSO_4_ 24 h	↑ Total AsA (roots)	↑ Reduced AsA (roots) ↓ DHA/AsA (n.s.)	↑ APX, GR (leaves)	[17]
	*Phaseolus vulgaris* (leaves)	2 μM CdSO_4_ 0, 24, 48, 72 h	↑ Total AsA	Long term (>48 h) ↑ DHA Long term (>48 h) ↑ DHA/AsA	↑ APX, GR	[46]
	*Phaseolus vulgaris*	0, 100 μM CdCl_2_ 4 days	↑ mRNA levels of GMP, GME, GalDH, GalLDH			[43]
	*Pinus sylvestris*	0, 5, 50 μM CdSO_4_ 6, 12, 24, 48, 96 h	Transient ↑ total AsA followed by ↓ (50 μM Cd)	Transient ↑of reduced AsA followed by ↓ (>24 h)	↑ MDHAR Ttransient ↓ APX followed by ↑ APX (>24 h)	[47]
	*Ceratophyllum demersum*	10 μM CdCl_2_ 1 week	↓ Total AsA	↓ Reduced AsA DHA > reduced AsA ↑ DHA/AsA	↑ APX (2x) ↓ MDHAR, DHAR	[48]
	*Brassica juncea* (shoots)	0, 50, 200 μM Cd(NO_3_)_2_ 7 days	↓ Total AsA ( <controls) (200 μM Cd)	↑ Reduced AsA (50 μM Cd) ↓ Reduced AsA ( <controls) (200 μM Cd)		[49]
**Cadmium (Cd)**	*Brassica juncea* (roots)	0, 50, 200 μM Cd(NO_3_)_2_ 7 days	↑ Total AsA (50 μM Cd) ↓ Total AsA ( >controls) (200 μM Cd)	↑ Reduced AsA (50 μM Cd) ↓ Reduced AsA ( >controls) (200 μM Cd)		[49]
	*Bechmeria nivea Gaud*	0, 1, 3, 7 mg/L Cd 1, 2, 3, 7, 10 days		↑ Reduced AsA followed by ↓ at high Cd concentration ↑ DHA ↑ DHA/AsA	↑ APX, GR	[50]
	*Shorea robusta*	1 mg/L, 5mg/L, 10 mg/L CdCl_2_ 4 months		↑ AsA		[51]
**Aluminum (Al)**	*Oryza Sativa*	0, 80, 160 μM Al_2_(SO_4_)_3_ 0, 5, 10, 15, 20 days	↓ Total AsA	↓ Reduced AsA ↑ DHA/AsA	↑ APX, MDHAR, DHAR, GR	[52]
	*Nicotiana tabacum* SR-1 wildtype	0, 400 μM AlCl_3_ 24 h	↑ Total AsA	↑ DHA ↑ DHA/AsA	↑ APX	[53]
	*Nicotiana tabacum* overexpressing *Arabidopsis thaliana* cytosolic DHAR (DHAR-OX)	0, 400 μM AlCl_3_ 24 h	↑ Total AsA	↑ Reduced AsA ↓ DHA ↓ DHA/AsA	↑ APX	[53]
**Arsenic (As)**	*Shorea robusta*	1 mg/L, 5mg/L, 10 mg/L As_2_O_3_ 4 months		↑ AsA		[51]
**Lead (Pb)**	*Shorea robusta*	1 mg/L, 5mg/L, 10 mg/L Pb(C_2_H_3_O_2_)_2_·3H_2_O 4 months		↑ AsA		[51]
**ESSENTIAL MICRONUTRIENTS**						
**Copper (Cu)**	*Arabidopsis thaliana*	2, 5 μM CuSO_4_ 24 h	↓ Total AsA (roots) ↑ Total AsA (leaves)	↑ DHA/AsA (roots)	↑ APX (roots) ↑ GR (leaves)	[26]
	*Arabidopsis thaliana*	10 μM CuSO_4_ 24 h	↓ Total AsA (roots) ↑ Total AsA (leaves)	↓ Reduced AsA, DHA (roots) ↑ Reduced AsA (leaves)	↓ APX (roots)	[17]
	*Arabidopsis thaliana*	0, 5, 25, 50, 100 μM CuSO_4_ 1, 3, 7 days		Short term ↑ reduced AsA (1, 3 days) Short term ↑ DHA (3 days)	Short term ↑ DHAR Long term ↑ GR, MDHAR	[54]
	*Phaseolus vulgaris* (roots)	15 μM CuSO_4_ 1, 5, 24, 48, 72, 96, 120, 168 h	↑ Total AsA	↑ Reduced AsA ↑ DHA ↑ DHA/AsA	Short term ↑ DHAR Long term slightly ↓ DHAR ↑ GR Long term ↑ MDHAR, APX	[55]
	*Phaseolus vulgaris* (leaves)	50 μM CuSO_4_ 0, 24, 48, 72, 96, 120, 144, 168 h	↑ Total AsA	↑ Reduced AsA Short term ↓ DHA followed by transient ↑ DHA Long term ↓ DHA ↓ DHA/AsA	↑ MDHAR, GR Long term ↑ APX, DHAR	[56]
	*Oryza Sativa* (MSE-9)	0, 10, 50, 100 μM CuSO_4_ 1, 5 days	↑ Total AsA		↑ APX Long term ↑ GR (>50 μM Cu)	[57]
	*Morus alba* L. cv. Kanva-2	0.0, 0.1, 1.0, 100 μM CuSO_4_ 25, 50 days	↑ Total AsA	↑ DHA (Cu-deficient,-excess) ↑ DHA/AsA (Cu-deficient,-excess)	↑ APX, GR	[58]
	*Hordeum vulgare* L. cv. Obzor	15, 150, 1500 μM CuSO_4_ 5 days	↑ Total AsA	↓ % reduced AsA		[59]
**Znic (Zn)**	*Phaseolus vulgaris* (roots)	50 μM ZnSO_4_ 1, 5, 24, 48, 72, 96, 120, 144, 168 h	0–120 h ↓ Total AsA >120 h ↑ Total AsA	1–96 h ↓ reduced AsA >96 h ↑ reduced AsA >72 h ↑ DHA ↑ DHA/AsA	1–96 h ↑ APX	[60]
	*Phaseolus vulgaris* (leaves)	50 μM ZnSO_4_ 1, 5, 24, 48, 72, 96, 120, 144, 168 h	↑ Total AsA	↑ Reduced AsA 48 h, >96 h ↑ DHA >96 h ↑ DHA/AsA	↑ DHAR Long term ↑ MDHAR, APX, GR	[60]
	*Cajanus cajan* LRG30 (long duration type)	2.5, 5.0, 7.5 mM ZnSO_4_·7H_2_O germinate for 6 days		↓ AsA	↑ GR	[61]
	*Cajanus cajan* ICPL87 (short duration type)	2.5, 5.0, 7.5 mM ZnSO_4_·7H_2_O germinate for 6 days		↓ AsA	↑ GR	[61]
	*Brassica juncea*	0.007, 0.05, 5.0, 10 mM ZnSO_4_ 10 days	↑ Total AsA		↑ MDHAR, DHAR, APX, GR	[62]
**Nickel (Ni)**	*Oryza sativa*	200, 400 μM NiSO_4_ 0, 5, 10, 15, 20 days		↑ Reduced AsA ↑ DHA ↑ DHA/AsA	↑ MDHAR, APX, GR	[63]
	*Cajanus cajan* LRG30 (long duration type)	0.5, 1.0, 1.5 mM NiSO_4_·6H_2_O germinate for 6 days		↓ AsA	↑ GR	[61]
	*Cajanus cajan* ICPL87 (short duration type)	0.5, 1.0, 1.5 mM NiSO_4_·6H_2_O germinate for 6 days		↓ AsA	↑ GR	[61]
**Manganese (Mn)**	*Vigna unguiculata* cv. TVu 91	0.2, 50, 100 μM MnSO_4_ 6 days		↓ Reduced AsA ↓ AsA/total AsA		[64]
	*Vigna unguiculata* cv. TVu1987	0.2, 50, 100 μM MnSO_4_ 6 days			↑ MDHAR, GR	[64]
	*Hordeum vulgare* L. cv. Obzor	183, 1830, 18300 μM MnCl_2_ 5 days	Transient ↑ total AsA followed by ↓		↓ APX	[59]
	*Oryza sativa* L. cv. Pant-12	3, 6 mM MnCl_2_ 0, 10, 20 days		↓ Reduced AsA ↓ DHA ↑ DHA/AsA	↑ MDHAR, DHAR, GR, APX	[65]

Abbrevations: AsA, ascorbate; DHA, dehydroascorbate; DHAR, dehydroascorbate reductase; MDHAR, monodehydroascorbate reductase; APx, ascorbate peroxidase; GR, glutathione reductase, DHAR-OX, DHAR overexpressing, n.s., not significant.
